# Association of the Timing and Type of Acute Symptomatic Seizures With Poststroke Epilepsy and Mortality

**DOI:** 10.1161/STROKEAHA.124.050045

**Published:** 2025-04-24

**Authors:** Kai Michael Schubert, Dominik Zieglgänsberger, Giulio Bicciato, Laura Abraira, Estevo Santamarina, José Álvarez-Sabín, Carolina Ferreira-Atuesta, Mira Katan, Lucia Sinka, Robert Terziev, Nico Deligas, Barbara Erdélyi-Canavese, Ansgar Felbecker, Philip Siebel, Michael Winklehner, Tim J. von Oertzen, Judith N. Wagner, Gian Luigi Gigli, Annacarmen Nilo, Francesco Janes, Giovanni Merlino, Mariarosaria Valente, María Paula Zafra-Sierra, Luis Carlos Mayor-Romero, Julian Conrad, Stefan Evers, Matias Alet, Kazuki Fukuma, Masafumi Ihara, Benjamin Landau, Piergiorgio Lochner, Frauke Roell, Francesco Brigo, Carla Bentes, Ana Rita Peralta, Teresa Pinho e Melo, Mark R. Keezer, John S. Duncan, Josemir W. Sander, Barbara Tettenborn, Matthias J. Koepp, Marian Galovic

**Affiliations:** Department of Neurology, Clinical Neuroscience Center, University Hospital and University of Zurich, Switzerland (K.M.S., G.B., M.K., L.S., R.T., M.G.).; Department of Neurology with Experimental Neurology, Charité - Universitätsmedizin Berlin, Germany (R.T.).; Epilepsy Unit, Department of Neurology, Vall d’Hebron Hospital Universitari, Barcelona; Universitat Autonoma de Barcelona, Bellaterra, Spain (L.A., E.S., J.A.-S.).; Department of Neurology, Kantonsspital St. Gallen, Switzerland (D.Z., N.D., B.E.-C., A.F., P.S., B.T.).; Department of Clinical & Experimental Epilepsy, UCL Queen Square Institute of Neurology, London; Chalfont Centre for Epilepsy, Chalfont St Peter, United Kingdom (C.F.-A., J.S.D., J.W.S., M.J.K., M.G.).; Department of Neurology, Icahn School of Medicine at Mount Sinai, New York, NY (C.F.-A.).; Johannes Kepler University Linz, Kepler University Hospital, Department of Neurology, Austria (T.J.O., J.N.W., M.W.).; Department of Medicine, University of Udine and Clinical Neurology, Udine University Hospital, Italy (G.L.G., A.N., F.J., G.M., M.V.).; Department of Neurology, Fundación Santa Fe de Bogotá, Universidad de Los Andes, Universidad del Bosque, Colombia (M.P.Z.-S., L.C.M.-R.).; Department of Neurology, University of Muenster, Germany (J.C., S.E.).; Division for Neurodegenerative Diseases, Department of Neurology, Universitaetsmedizin Mannheim, University of Heidelberg, Germany (J.C.).; Department of Neurology, Krankenhaus Lindenbrunn, Coppenbrügge, Germany (S.E.).; Centro Integral de Neurología Vascular, Fleni, Ciudad Autónoma de Buenos Aires, Argentina (M.A.).; Department of Neurology, National Cerebral and Cardiovascular Center, Osaka, Japan (K.F., M.I.).; Department of Neurology, Saarland University Medical Center, Homburg, Germany (B.L., P.L., F.R.).; Department of Neurology, Hospital of Merano (SABES-ASDAA), Italy (F.B.).; Stichting Epilepsie Instellingen Nederland–(SEIN), Heemstede, the Netherlands (M.R.K., J.W.S.).; Department of Neurosciences and Mental Health (Neurology), Hospital de Santa Maria-CHULN; Centro de Estudos Egas Moniz, Faculdade de Medicina, Universidade de Lisboa, Portugal (C.B., A.R.P., T.P.M.).; Centre Hospitalier de l’Université de Montréal, Canada (M.R.K.).; Specialist Clinic for Neurorehabilitation, Kliniken Beelitz, Beelitz-Heilstätten, Germany (N.D.).; Department of Neurology, University Hospital and University of Basel, Switzerland (M.K.).; Department of Neurology, Evangelisches Klinikum Gelsenkirchen, Academic Hospital University Essen-Duisburg, Germany (J.N.W.).; Department of Neurology, West China Hospital, Sichuan University, Chengdu, China (J.W.S.).; Department of Neurology, Schulthess Klinik, Zurich, Switzerland (L.S.).

**Keywords:** epilepsy, ischemic stroke, seizures, status epilepticus, stroke

## Abstract

**BACKGROUND:**

Acute symptomatic seizures (ASyS) increase the risk of epilepsy and mortality after a stroke. The impact of the timing and type of ASyS remains unclear.

**METHODS:**

This multicenter cohort study included data from 9 centers between 2002 and 2018, with a final analysis in February 2024. The study included 4552 adults (2005 female; median age, 73 years) with ischemic stroke and no seizure history. Seizures were classified using International League Against Epilepsy definitions. We examined ASyS occurring within 7 days after stroke. The main outcomes were all-cause mortality and epilepsy. Validation of the updated SeLECT score (SeLECT-ASyS) was performed in 3 independent cohorts (Switzerland, Argentina, and Japan) collected between 2012 and 2024, including 74 adults with ASyS.

**RESULTS:**

The 10-year risk of poststroke epilepsy ranged from 41% to 94%, and mortality from 36% to 100%, depending on ASyS type and timing. ASyS on stroke onset day had a higher epilepsy risk (adjusted hazard ratio [aHR], 2.3 [95% CI, 1.3–4.0]; *P*=0.003) compared with later ASyS. Status epilepticus had the highest epilepsy risk (aHR, 9.6 [95% CI, 3.5–26.7]; *P*<0.001), followed by focal to bilateral tonic-clonic seizures (aHR, 3.4 [95% CI, 1.9–6.3]; *P*<0.001). Mortality was higher in those with ASyS presenting as focal to bilateral tonic-clonic seizures on day 0 (aHR, 2.8 [95% CI, 1.4–5.6]; *P*=0.004) and status epilepticus (aHR, 14.2 [95% CI, 3.5–58.8]; *P*<0.001). The updated SeLECT-ASyS model, available as an application, outperformed a previous model in the derivation cohort (concordance statistics, 0.68 versus 0.58; *P*=0.02) and in the validation cohort (0.70 versus 0.50; *P*=0.18).

**CONCLUSIONS:**

ASyS timing and type significantly affect epilepsy and mortality risk after stroke, improving epilepsy prediction and guiding patient counseling.

Stroke is a major cause of epilepsy in older adults, contributing to over half of new-onset epilepsy cases in individuals aged ≥65 years.^1^ Poststroke seizures are associated with an increased risk of mortality, poor functional outcomes, disability, and dementia.^2,3^

Poststroke seizures are categorized into acute symptomatic seizures (ASyS), occurring within the first 7 days after a stroke, and remote symptomatic seizures (RSyS), which are unprovoked seizures occurring later.^4^ ASyS are considered provoked and do not qualify as epilepsy. In contrast, a single or multiple RSyS following ischemic stroke fulfills the International League Against Epilepsy (ILAE) practical definition of epilepsy due to a heightened, >60% recurrence risk of seizures.^5^

ASyS is a major risk factor for epilepsy and mortality following ischemic stroke.^3,6^ Recent research underscores the heterogeneity among ASyS, suggesting certain subtypes confer higher risks than others.^7^ We have shown that ASyS presenting as status epilepticus carries a markedly elevated risk of epilepsy and mortality compared with short ASyS after ischemic stroke.^3^ However, there remains a knowledge gap about other characteristics of ASyS that may be associated with an increased risk of seizures or unfavorable outcomes.

We hypothesized that the timing and type of a short ASyS influence the risk of poststroke epilepsy (PSE) and mortality. We assessed this hypothesis using data from a large multicenter registry of poststroke seizures. We implemented this knowledge in an updated prognostic model that improves the prediction of epilepsy following ASyS after ischemic stroke.

## Methods

The data that support the findings of this study are available from the corresponding author upon reasonable request.

### Participants

We analyzed both a derivation and a validation cohort of participants. The derivation cohort was drawn from a multicenter registry established as part of the SeLECT study,^6^ consisting of 9 international cohorts (n=4552) of adults with neuroimaging-confirmed acute ischemic stroke. We added 3 additional independent international cohorts of people with ASyS following ischemic stroke as a validation data set by directly approaching investigators with an interest in PSE research. The validation cohort included participants with ASyS following ischemic stroke from 3 additional independent cohorts. We excluded individuals with transient ischemic attacks, a history of seizures or epilepsy, primarily hemorrhagic stroke (eg, primary intracerebral hemorrhage or primary subarachnoid hemorrhage), reinfarction during follow-up, or potentially epileptogenic comorbidities (such as intracranial tumors, cerebral venous thrombosis, severe traumatic brain injury [Glasgow Coma Scale ≤8; loss of consciousness >24 hours, posttraumatic amnesia >7 days, and significant brain injury on imaging], or prior brain surgery) while retaining patients with a primary ischemic stroke and secondary hemorrhagic transformation. Detailed descriptions of the individual cohorts are provided in the Supplemental Material.

Informed consent, obtained either in written or verbal form (4 cohorts utilized written consent, while 2 cohorts used both written and verbal consent), was acquired. In 3 cohorts consent requirements were waived by the regulatory authorities, as outlined in the Supplemental Material.

### Definitions

According to ILAE recommendations, seizures were classified as ASyS (occurring within 7 days after stroke) or RSyS (spontaneous unprovoked seizures >7 days after stroke).^4^ The occurrence of an RSyS was categorized as PSE due to its high seizure recurrence risk, exceeding the 60% risk required for the ILAE pragmatic definition of epilepsy.^5,8^ Status epilepticus was classified according to the revised ILAE definition.^9^ Electrographic status epilepticus was defined according to Salzburg and revised American Clinical Neurophysiology Society criteria.^10,11^ ASyS not qualifying as status epilepticus were defined as short ASyS and classified into subtypes (focal aware, focal with impaired awareness, focal to bilateral tonic-clonic, or undetermined) based on the current ILAE nomenclature.^12^ We dichotomized the timing of ASyS into those occurring on the same day as stroke onset (day 0) versus those occurring later because the majority of ASyS occurred on day 0. In individuals with multiple ASyS, we only considered the first reported ASyS. Further definitions are detailed in the eMethods in the Supplemental Material.

### Statistical Analysis

First, we used multivariable Cox proportional hazards regression to assess the relationship between the type and timing of ASyS and the time to PSE or death, while adjusting for covariates (age, sex, National Institutes of Health Stroke Scale score at admission, cortical involvement, involvement of the middle cerebral artery territory, stroke cause, reperfusion treatment, and antiseizure medication treatment after ASyS). Cases were censored at the time of death, first RSyS, or last follow-up. Adjusted risk estimates for PSE or death were obtained from these multivariable Cox regression models. The proportional hazards assumption was assessed using Schoenfeld residuals. In cases where time-varying effects were suggested, complementary analyses were conducted using an accelerated failure time model (Table S8).

Second, we compared the performance of a previously described prognostic model for PSE (SeLECT_2.0_)^3^ in stroke survivors with versus without ASyS using concordance statistics (C statistics). We also compared the observed risk of seizures in those with versus without ASyS having similar SeLECT_2.0_ score strata (3–4 points and 5–6 points) using Kaplan-Meier estimate plots and log-rank tests.

Next, we updated the existing SeLECT_2.0_ model, specifically tailoring it for stroke survivors with ASyS to improve the prediction of PSE in this particularly vulnerable population. Least Absolute Shrinkage and Selection Operator Cox regression was used initially for variable selection, utilizing a penalty function to refine the model.^13^ The regularization parameter lambda in the Least Absolute Shrinkage and Selection Operator regression was selected using k-fold cross-validation (k=10). Following the Least Absolute Shrinkage and Selection Operator selection, we used Wald stepwise backward regression as an additional method to identify significant variables and compare them with those selected by Least Absolute Shrinkage and Selection Operator. To account for death as a competing risk, we also used competing risk regression based on Fine and Gray’s subdistribution hazard model, which allowed us to calculate the cumulative incidence function for late seizures (see Table S4). We assigned integer values to the retained variables based on their adjusted hazard ratio (aHR; see Table S5) to calculate a clinical risk score for each study individual.

To evaluate the updated model’s discrimination, that is, the ability to distinguish between high- and low-risk cases, we estimated the C statistics (95% CI). Recognizing that prognostic models derived from multivariable regression can exhibit optimism and potentially overestimate predictions when applied to new patient cohorts,^14^ we introduced a shrinkage factor. This factor was estimated through 1000 bootstrapped random samples to adjust the C statistics for overoptimism, a technique previously used to enhance model generalizability.^15,16^ We also assessed model calibration, that is, the agreement between predicted and observed risks, using calibration plots (see Figure S5). Perfect calibration is represented by a 45° diagonal line, whereas relevant deviation above or below this line reflects under or overprediction. We used a leave-one-cohort-out strategy for cross-validation of model performance.

Lastly, we computed the change of occurrence of a seizure in the next year, a parameter that may be relevant for assessing the fitness to drive in people with seizures, using the standard statistical definition of conditional risks^17^ as detailed previously^32^ (see Figure S2). Previously proposed change of occurrence of a seizure in the next year thresholds are <20% to 40% for private driving and <2% for professional driving,^18–20^ although these may differ based on local regulations.

All analyses were conducted using R Statistical Software, version 4.0.3, and SPSS, version 26 (IBM), and followed the STROBE guidelines (Strengthening the Reporting of Observational Studies in Epidemiology; Supplemental Material).

## Results

**Figure 1.**
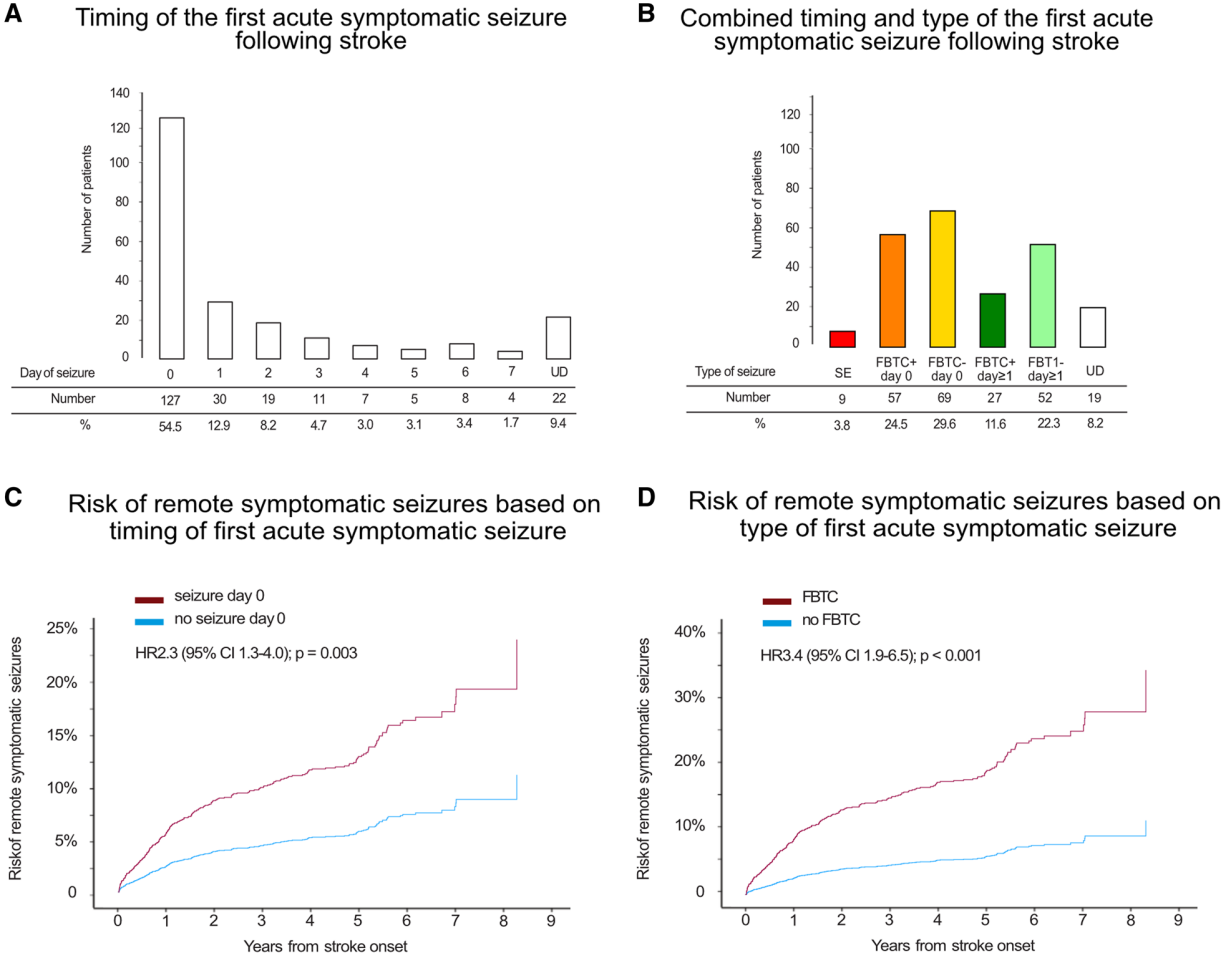
**Characterization of acute symptomatic seizures (ASyS) timing and type. A** and **B**, The distribution and stratification of the first ASyS occurring after ischemic stroke. **A** illustrates the timing of ASyS, while **B** combines the timing (day 0 vs other days) with the type of seizure (eg, focal to bilateral tonic-clonic seizure [FBTCS], status epilepticus [SE]). The color coding in **B** aligns with the stratification of ASyS shown in Figure 2. Due to the small number of cases, SE and UD (undetermined/unknown seizure timing) seizures were not further differentiated by timing. **C** and **D**, Kaplan-Meier estimates (n=4552) of the time to poststroke epilepsy stratified by ASyS timing (day 0 vs day≥1; **B**) and type (FBTCs vs other short seizure type; **C**). HR indicates hazard ratio.

The study included 4552 individuals from 9 centers. Their baseline characteristics are shown in Table S1. ASyS occurred in 5% (n=233) of participants. The first ASyS was presented on the same day as the stroke (day 0) in 55% (n=127; Figure 1A; Table S2). The frequency of ASyS stratified by type and timing is shown in Figure 1B.

### Risk of PSE

ASyS on day 0 had a higher risk of PSE (aHR, 2.3 [95% CI, 1.3–4.0]; *P*=0.003; Figure 1C; Table 1) compared with ASyS occurring later after stroke. Other ASyS timing cutoffs did not yield relevant differences in the risk of PSE (Figure S2). Regarding seizure types, ASyS presenting as status epilepticus had the highest risk of PSE (aHR, 9.6 [95% CI, 3.5–26.7]; *P*<0.001), followed by focal to bilateral tonic-clonic seizure (FBTCS; aHR, 3.4 [95% CI, 1.9–6.3]; *P*<0.001; Figure 1D; Table 1) compared with other ASyS types (Figure S4). The full results of the multivariable model and other variables independently associated with PSE (stroke severity, location, and cause) are shown in Table 1.

**Table 1.**
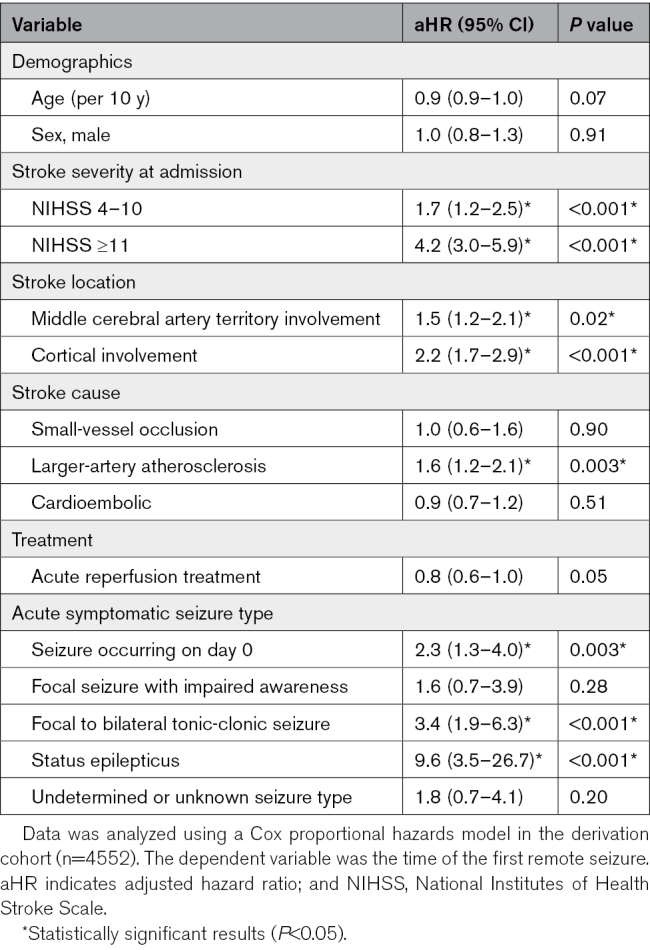
Multivariable Cox Regression Model of Time to First Remote Symptomatic Seizure

**Figure 2.**
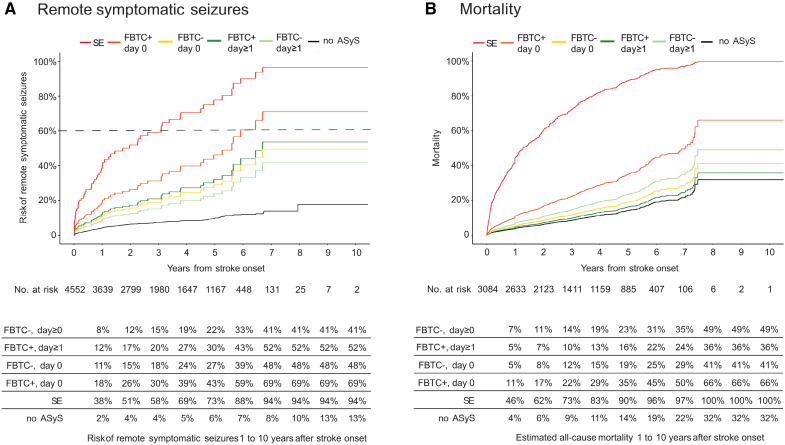
**Risk of poststroke epilepsy or mortality following acute symptomatic seizures (ASyS) after stroke.** Data according to the type and timing of ASyS and association with remote symptomatic seizure (RSyS; n=4552; **A**) and mortality (n=3084; **B**). The tables below each graph display the Kaplan-Meier estimates of the risk of RSyS 1 to 10 years after index stroke according to the type of ASyS. All results were obtained after adjusting for covariates (age, sex, National Institutes of Health Stroke Scale score at admission, cortical involvement, involvement of the middle cerebral artery territory, stroke cause, reperfusion treatment, and antiseizure medication treatment after ASyS). The dotted line denotes the 60% cutoff for the risk of unprovoked seizures used in the International League Against Epilepsy practical clinical definition of epilepsy. FBTCS indicates focal to bilateral tonic-clonic seizure; and SE, status epilepticus.

Stroke survivors with a non-FBTC short ASyS occurring on day 1 or later after stroke had a 41% risk of developing PSE 10 years after stroke, compared with a 69% risk in those having a FBTC short ASyS on day 0 after stroke and a 94% risk in those with acute symptomatic status epilepticus (Figure 2A; Table S2). The 10-year risk of PSE was 13% in stroke survivors without ASyS.

### Mortality

A higher risk of all-cause mortality was observed in those with ASyS presenting as FBTCS on day 0 (aHR, 2.8 [95% CI, 1.4–5.6]; *P*=0.004) and those with acute symptomatic status epilepticus (aHR, 14.2 [95% CI, 3.5–58.8; *P*<0.001). Other subtypes of ASyS were not associated with mortality (Table 2).

**Table 2.**
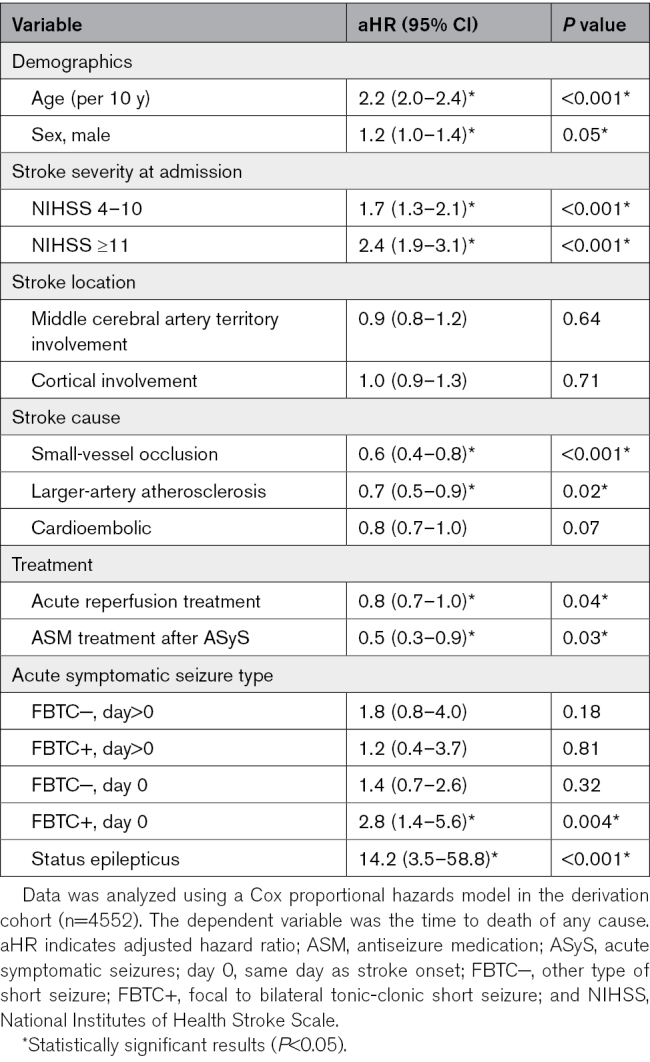
Multivariable Cox Regression Model of Time to Death

Individuals with a stroke and a non-FBTC short ASyS occurring on day 1 or later after stroke had a 49% risk of all-cause mortality 10 years after stroke, compared with a 66% risk in those having a FBTC short ASyS on day 0 after stroke and a 100% risk in those with acute symptomatic status epilepticus (Figure 2B). The overall 10-year risk of all-cause mortality was 32% in those without ASyS.

### Prognostic Modeling

**Figure 3.**
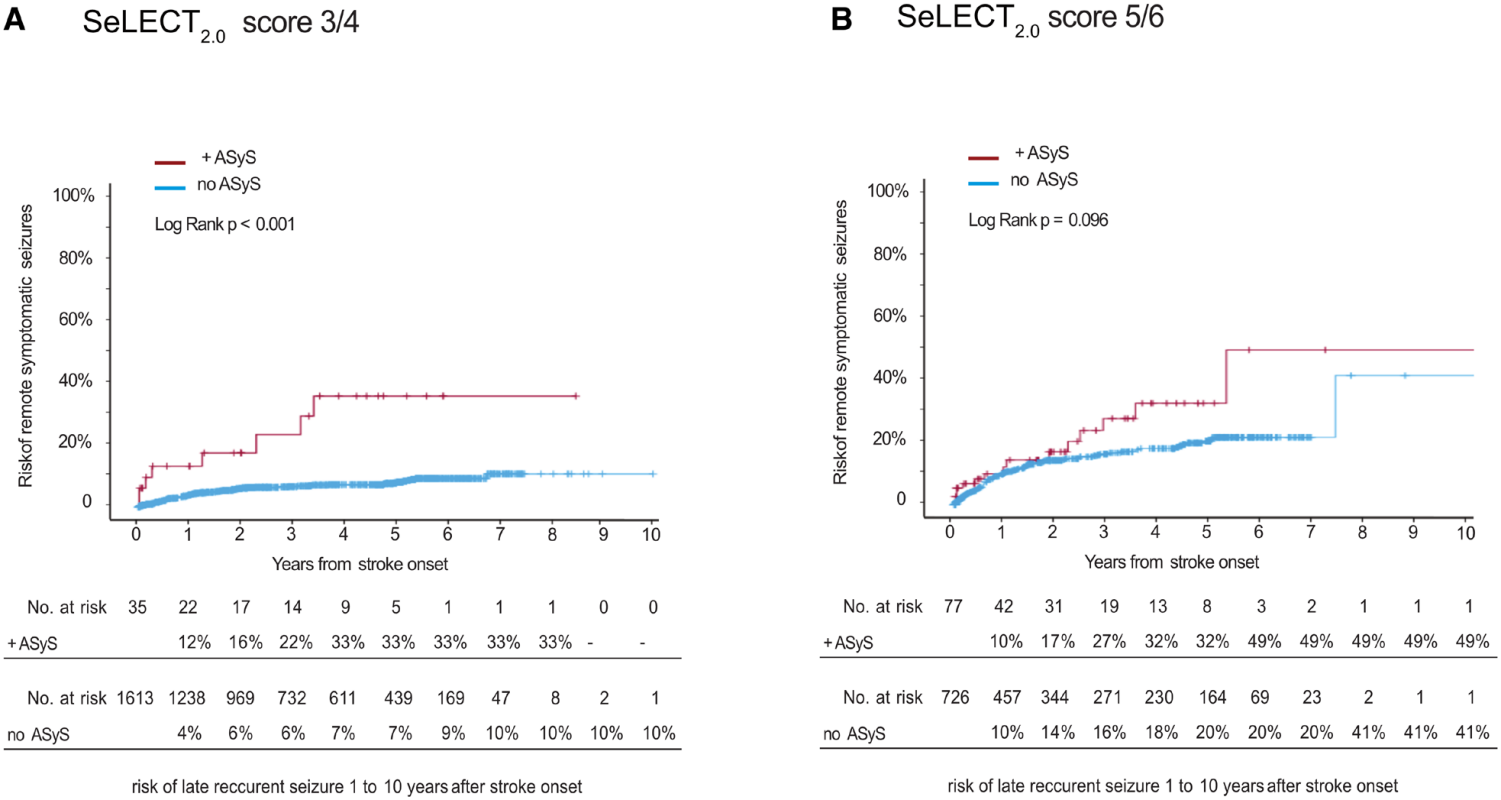
**Risk of epilepsy in individuals with or without acute symptomatic seizures (ASyS) having a similar SeLECT_2.0_ score.** Kaplan-Meier estimates (n=4552) of the time to poststroke epilepsy in those with a SeLECT_2.0_ score of 3 to 4 (**A**) or 5 to 6 (**B**) points. Separate curves are shown for individuals with (red) or without (blue) ASyS. Those with a SeLECT_2.0_ score of 3 to 4 who suffered ASyS had a higher risk of poststroke epilepsy (higher risk of remote symptomatic seizures) compared with those without ASyS (*P*<0.001). There was a similar but nonsignificant trend in individuals with a SeLECT_2.0_ score of 5 to 6 who had ASyS compared with those without (*P*=0.096). ASyS is defined as seizures occurring within the first 7 days following ischemic stroke.

We observed that the performance of a previously published prognostic model predicting the risk of PSE (SeLECT_2.0_) was low in individuals with ASyS (C statistics, 0.58 [95% CI, 0.49–0.67]; n=233) compared with a better performance in the overall cohort (C statistics, 0.75 [95% CI, 0.72–0.78]; n=4552). The observed risk of PSE in stroke survivors with ASyS was higher compared with those without ASyS who had a similar SeLECT_2.0_ score (Figure 3; SeLECT_2.0_ score 3 to 4, *P*<0.001; SeLECT_2.0_ score 5 to 6, *P*=0.10).

Thus, the SeLECT_2.0_ model may not adequately capture the risk of epilepsy in those with ASyS. To overcome these limitations, we updated the SeLECT_2.0_ model specifically for those having ASyS after stroke and to implement the above findings on the risk of epilepsy according to ASyS type and timing.

We selected predictors using stepwise backward elimination of a Cox regression model in stroke survivors with ASyS (Table S4). The variables retained in the final model were timing and type of first ASyS, large-vessel atherosclerotic stroke pathogenesis stratified by sex, and stroke involving the cerebral cortex. The calculation of the new model termed SeLECT-ASyS and ranging from 0 to 7 points, is shown in Table 3. The SeLECT-ASyS model had better discrimination for time to PSE compared with the original SeLECT_2.0_ model (C statistics, 0.68 [95% CI, 0.61–0.76] versus 0.58 [95% CI, 0.49–0.67]; *P*=0.02) in stroke survivors with ASyS. SeLECT-ASyS demonstrated better, near-optimal calibration for long-term outcomes compared with the less optimal calibration of the SeLECT_2.0_ model in those with ASyS, although the calibration curves indicated less accuracy for predicting the 1-year occurrence of PSE and lacked data for lower probability outcomes (Figure S5). We cross-validated the results using a leave-one-cohort-out strategy (Table S6). To further support our findings, we evaluated a validation cohort of 74 adults with ASyS following ischemic stroke who met the eligibility criteria (Switzerland, n=32; Argentina, n=23; and Japan, n=19). The baseline characteristics of these individuals are presented in Table S7. In this validation cohort, the SeLECT-ASyS model demonstrated superior discrimination with a C statistics of 0.70 (95% CI, 0.57–0.83) compared with the SeLECT_2.0_ model (C statistics, 0.59 [95% CI, 0.44–0.75]; *P*=0.18), indicating better predictive accuracy for RSyS (Figure S6).

**Table 3.**
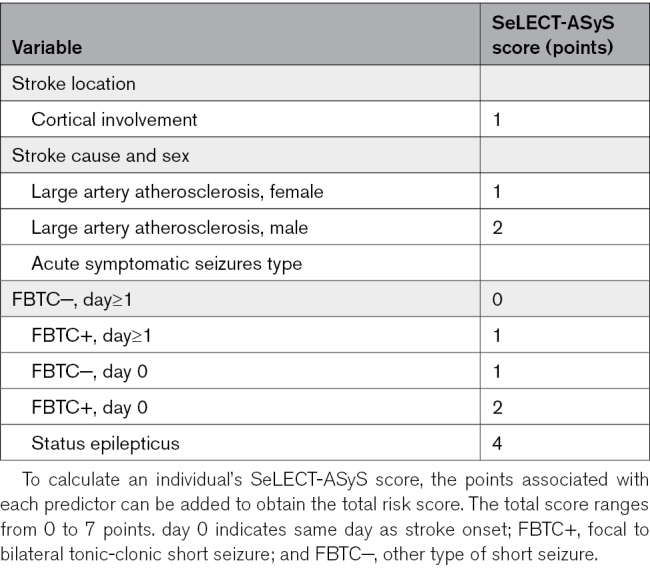
Calculation of the SeLECT-ASyS Score

**Figure 4.**
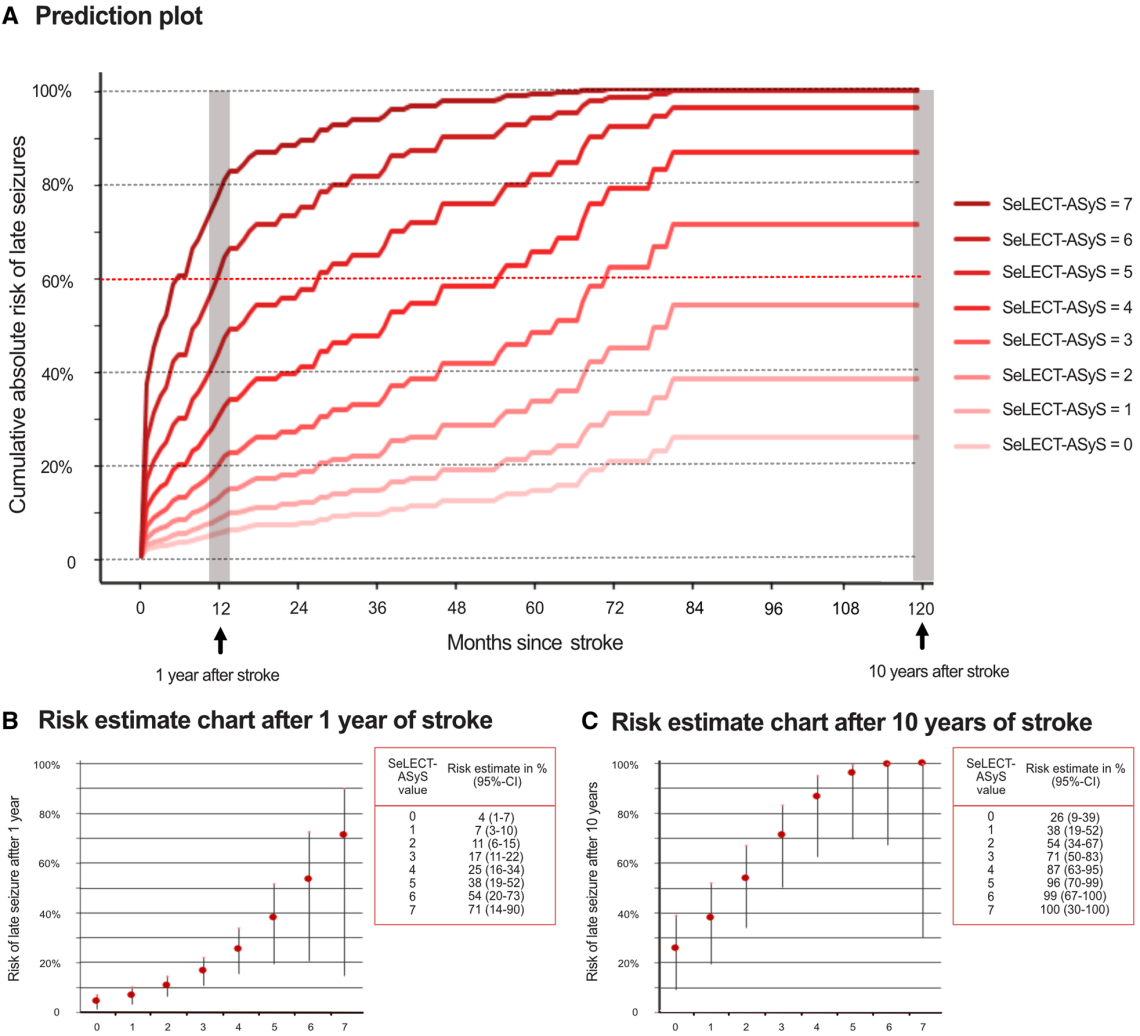
**Predicted risk of poststroke epilepsy according to a new prognostic model in stroke survivors with acute symptomatic seizures. A**, The predicted risk of poststroke epilepsy (unprovoked remote symptomatic seizures) is 0 to 120 months after stroke. Each curve represents the estimates for a SeLECT-ASyS value ranging from 0 to 6. Risk estimate charts of late seizures 1 year and 10 years after stroke according to SeLECT-ASyS score values are displayed in **B** and **C**, respectively. Vertical lines indicate 95% CIs.

The lowest score SeLECT-ASyS value (0 points) predicts a 26% risk (95% CI, 9–39) of PSE 10 years following a stroke, compared with a 100% risk (95% CI, 30–100) for the highest value (7 points; Figure 4). A comparison to the SeLECT_2.0_ values for patients without ASyS is shown in Figure S1. In addition, we updated the values for change of occurrence of a seizure in the next year (Figure S2), a parameter that may be helpful for assessing the risks of safe driving. The new SeLECT-ASyS model was implemented in the SeLECT score smartphone applications available for iOS and Android and the web-based calculator.

### Real-World Case Example

A male patient in his 70s presented with an acute ischemic stroke in the right middle cerebral artery territory in 2021, confirmed by neuroimaging. The stroke had cortical involvement and was classified as atherosclerotic in origin (TOAST [Trial of ORG 10172 in Acute Stroke Treatment] classification – type 1). Clinically, he exhibited moderate to severe left-sided hemiparesis, facial palsy, dysarthria, and dysphagia. His National Institutes of Health Stroke Scale score was 25 at the initial evaluation, reduced to 15 following reperfusion therapy.

On the day of admission (day 0), the patient experienced an FBTCS, observed by hospital staff. This event was categorized as an ASyS. Based on the SeLECT_2.0_ score, the patient’s calculated risk of PSE within 10 years was 30% (95% CI, 13–43) with a total score of 5. However, the updated SeLECT-ASyS model, tailored specifically for individuals with ASyS, also assigned a score of 5 but predicted a markedly higher 96% (95% CI, 70–99) risk of PSE within 10 years.

Initial treatment included Levetiracetam (500 mg twice daily), which was discontinued upon discharge as the seizure was deemed acute symptomatic. Four months postdischarge, the patient reported recurrent brief episodes of tremors in the left hand, consistent with simple focal seizures. A diagnosis of PSE was made, and Levetiracetam was restarted. Since resuming antiseizure medication, the patient has remained seizure-free.

## Discussion

In this study, we investigated the influence of both timing and type of ASyS on the risk of epilepsy and mortality after ischemic stroke. Our findings reveal substantial heterogeneity among ASyS, indicating that events occurring on the day of stroke onset (day 0) and those manifesting as FBTCS or status epilepticus had a higher risk of developing PSE. FBTCS on day 0 and acute symptomatic status epilepticus had a high, ≥69% risk of epilepsy and a ≥66% risk of mortality (Figure 2).

We also showed that a current state-of-the-art model for predicting PSE (SeLECT_2.0_) underperformed in stroke survivors with ASyS and confirmed this finding in independent validation cohorts. We implemented our results on the role of timing and types of ASyS in an updated model (SeLECT-ASyS), tailored specifically for stroke survivors experiencing ASyS. This model accurately captures the elevated risks of PSE in this subgroup and significantly outperforms the SeLECT_2.0_ model in those with ASyS. For individuals without ASyS, the SeLECT2.0 model remains the appropriate tool for risk prediction, ensuring that each model is applied within its intended population to optimize prognostic accuracy.

We previously showed that acute symptomatic status epilepticus is a predictor of mortality and epilepsy after stroke^3^ and discussed the potential explanations for this observation. The estimated risks for epilepsy and mortality in the current study are slightly higher compared with our previous study^3^ because of the completion of long-term (60-month) follow-up in the second largest cohort in the registry (Switzerland (2)) which results in more robust estimates. Building upon our prior research, we demonstrated in this study that ASyS presenting as FBTCS and occurring on day 0 also carry a higher risk of PSE and mortality. There are several potential explanations for this finding.

First, individuals experiencing ASyS early after stroke, that is, on day 0, and ASyS presenting as FBTCS may have a higher predisposition for generating seizures. This genetic or acquired vulnerability,^21^ conceptualized as a low seizure threshold,^22^ may predispose these individuals to an earlier onset of ASyS and the propagation of seizure activity across hemispheres. Such a low seizure threshold may also heighten the probability of subsequent RSyS during epileptogenesis.^22^

Second, the occurrence of ASyS on day 0 and a bilateral spread of seizures could be indicative of a more pronounced proepileptic impact of the stroke. Our previous research suggested that more severe strokes due to large artery atherosclerosis, affecting the middle cerebral artery territory, are more likely to result in PSE.^6^ But, the present study’s outcomes were adjusted for these variables, establishing independence from such factors. Other stroke characteristics warrant consideration. At a macroscopic level, the precise localization^23^ and connectivity of stroke may contribute to acute seizures and epileptogenesis. Strokes highly connected to the basal ganglia and cerebellum were found to be more likely to cause epilepsy.^24^ On a microscopic scale, cellular changes that are difficult to detect in vivo in humans, such as neurodegeneration, axonal and synaptic sprouting, blood-brain barrier damage, and inflammation,^21^ may promote acute seizures and epileptogenesis.

Third, disparities between ASyS occurring on day 0 and those manifesting later may be associated with the time-dependent dynamics of pathological mechanisms following a stroke, such as neuroinflammatory changes and metabolic derangements.^25^ Consequently, ASyS occurring early might be triggered by distinct mechanisms compared with those occurring later, potentially resulting in variations in the risk of PSE.

Lastly, ASyS may directly or indirectly contribute to epileptogenesis. While this concept has been consistently demonstrated in animal models of status epilepticus, it is less well-established for brief seizures.^26^ Some experimental evidence suggests that brief convulsive seizures may also contribute to the process of epileptogenesis.^26–28^

ASyS presenting as FBTCS on day 0 were independently associated with higher mortality after stroke. They may be a marker of significant macro or microscopic neuronal disruption caused by stroke, as discussed above. Furthermore, convulsive seizures have been linked to excitotoxic damage in animal models, potentially contributing to poor outcomes.^29^ Convulsive seizures may also be associated with injuries, heightened metabolic demand, and aspiration leading to pneumonia, further impacting overall outcomes.

To translate these findings into clinical practice, we incorporated them into an updated prognostic model. Initially, we demonstrated that the current state-of-the-art prognostic model for poststroke seizures, SeLECT_2.0_, underestimates the risk of PSE in the subset of stroke survivors with ASyS (Figure 3). Stroke survivors with ASyS represent a distinct group with unique predictors and a heightened risk of epilepsy compared with those without ASyS.^30^ Subsequently, we updated and validated the SeLECT_2.0_ model, resulting in SeLECT-ASyS, which exhibited superior discrimination and calibration compared with SeLECT_2.0_ in the ASyS subgroup. We also performed internal validation using optimism correction through bootstrapping and cross-validation (Table S6). Furthermore, we also confirmed SeLECT_2.0_’s underperformance in stroke survivors with ASyS in independent external validation cohorts. Consequently, SeLECT-ASyS emerges as the preferred model for prognostication in stroke survivors with ASyS.

The presented case example illustrates marked differences in estimated risks when utilizing SeLECT-ASyS as opposed to SeLECT_2.0_ for individuals with ASyS. These differences in risk are clinically meaningful and may have an impact on treatment considerations and the approach to follow-up in such cases. Risk estimates derived from the updated model consistently indicate moderate to high risks of PSE following ASyS (Figure 4). These risks are realistic, as corroborated by the favorable calibration of the model (Figure S5).

The most notable and practically relevant finding is a >60% 10-year risk of epilepsy in stroke survivors with ASyS presenting as FBTCS on day 0 or status epilepticus (28% of all ASyS cases; Figure 1B) and those with SeLECT-ASyS scores ≥3 points. This risk level aligns with the ILAE practical definition of epilepsy.^5^ But, it is crucial to acknowledge that this definition requires at least 1 unprovoked seizure occurrence and is, hence, not fully met in cases that suffered only an ASyS. Nevertheless, some clinicians may consider counseling these high-risk cases as if they had epilepsy, potentially recommending primary preventive treatment^31,32^ or extended follow-up. It is important to note, however, that the efficacy of primary preventive treatment after stroke remains unproven. Two ongoing antiepileptogenesis trials in stroke survivors may offer novel insights into the treatment of cases at high risk of PSE.^33,34^ If such treatments become available, the accurate prediction of the risk of PSE in those with ASyS will become central for the selection of candidates for antiepileptogenic medications.

Our study has several strengths. We assessed one of the largest multicenter cohorts of poststroke seizures. We translated our findings into a user-friendly prognostic model accessible through both smartphone and web applications. The updated model outperforms the current state-of-the-art model and its clinical significance was underscored through an illustrative case.

Our study has limitations. First, despite the inclusion of 9 international cohorts, enhanced statistical power, and generalizability of our results could be further achieved by incorporating data from North America, Asia, or Africa. Second, the practical constraints of performing continuous electroencephalograms in the entire multicenter registry limited our evaluation to clinically apparent seizures, not considering electrographic seizures. Third, the diagnosis of seizures relied largely on clinical observation, and systematic electroencephalogram monitoring was not performed in all cases. This approach may have resulted in an underestimation of seizures with subtle clinical signs. We did not consider purely electrographic events as seizures in this study. Future studies using continuous electroencephalogram monitoring should assess the impact of purely electrographic events on the risk of poststroke seizures. Fourth, data collected in the registry did not differentiate between seizures occurring immediately at stroke onset and those on the same day as the stroke. However, existing studies^35,36^ suggest that the majority of seizures on day 0 align with the immediate onset of the stroke. Fifth, our study lacked data on the discharge National Institutes of Health Stroke Scale score or National Institutes of Health Stroke Scale score assessed 72 hours posttreatment, which may more accurately predict the risk of poststroke seizures.^37^ Sixth, patients with ASyS may receive antiseizure medication treatment which may impact the risk of subsequent unprovoked seizures. To address this, we corrected all results for antiseizure medication treatment. Lastly, the SeLECT registry did not consistently collect data on the cause of death and long-term disability.

## Conclusions

We demonstrated varying mortality and epilepsy risks based on the type and timing of ASyS following stroke. We implemented these findings in an updated prognostic model (SeLECT-ASyS) that outperformed a previous model and is available as both a smartphone and web application. The 10-year epilepsy risk in those with ASyS presenting as FBTCS on day 0 or status epilepticus and those with SeLECT-ASyS≥3 points exceeded 60%, a cutoff used for the ILAE definition of epilepsy.^5^ These findings have the potential to inform counseling for stroke survivors with ASyS, particularly those with a high (>60%) risk for PSE.

## Article Information

### Acknowledgments

Drs Schubert, Zieglgänsberger, and Galovic conceptualized and designed the study. Drs Deligas, Erdélyi-Canavese, Felbecker, Siebel, Sinka, Nilo, Zafra-Sierra, Mayor-Romero, Alet, Fukuma, Ihara, Landau, Terziev, Bicciato, Ferreira-Atuesta, Katan, Abraira, Santamarina, Álvarez-Sabín, Winklehner, von Oertzen, Wagner, Gigli, Janes, Merlino, Valente, Conrad, Evers, Lochner, Roell, Brigo, Bentes, Peralta, Pinho e Melo, Tettenborn, Keezer, Koepp, Galovic, and J.W. Sander and J.S. Duncan contributed to the acquisition and analysis of the data. Drs Schubert and Galovic contributed to the interpretation of the data. Drs Schubert and Galovic drafted the article and figures. All coauthors revised the article for intellectual content.

### Sources of Funding

Dr Abraira is supported by the Fundació La Marató de TV3 (Spain). J.S. Duncan, J.W. Sander, and Dr Koepp are based at the National Institute for Health and Care Research University College London Hospitals Biomedical Research Centre, which is funded by the UK Department of Health. J.W. Sander holds an endowed position from the UK Epilepsy Society and receives additional research support from the Marvin Weil Epilepsy Research Fund and the Christelijke Vereniging voor de Verpleging van Lijders aan Epilepsie (the Netherlands). Dr Katan receives funding from the Swiss National Science Foundation.

### Disclosures

Dr Abraira reports travel support from Angelini Pharma; compensation from Union Chimique Belge for consultant services and Jazz Pharmaceuticals for other services; and travel support from Jazz Pharmaceuticals. Dr von Oertzen reports grants from the Austrian Science Fund; and compensation from UCB GmBH for other services. Dr Wagner reports compensation from Union Chimique Belge, Pfizer, and Janssen Cilag (Europe, Middle East, and Africa) for other services; compensation from Janssen Cilag EMEA for consultant services; compensation from Boehringer Ingelheim for other services; travel support from Boehringer Ingelheim, Novartis, and Janssen Cilag (Europe, Middle East and Africa); compensation from AstraZeneca for other services; and Novartis for consultant services. J.S. Duncan reports grants from Wellcome Trust. J.W. Sander reports compensation from The *Lancet*’s International Advisory Board for other services. Dr Koepp reports compensation from Matthias & Ronit Pressler-Koepp for consultant services and stock options in PrevEp. Dr Galovic reports compensation from Angelini Pharma, Eisai, and Union Chimique Belge for consultant services. The other authors report no conflicts.

### Supplemental Material

Supplemental Methods

Tables S1–S8

Figures S1–S6

STROBE Checklist

## References

[1] Hauser WA Annegers JF Kurland LT. Incidence of epilepsy and unprovoked seizures in Rochester, Minnesota: 1935-1984. Epilepsia. 1993;34:453–468. doi: 10.1111/j.1528-1157.1993.tb02586.x 8504780 10.1111/j.1528-1157.1993.tb02586.x

[2] Misra S Kasner SE Dawson J Tanaka T Zhao Y Zaveri HP Eldem E Vazquez J Silva LS Mohidat S. Outcomes in patients with poststroke seizures: a systematic review and meta-analysis. JAMA Neurol. 2023;80:1155–1165. doi: 10.1001/jamaneurol.2023.3240 37721736 10.1001/jamaneurol.2023.3240 PMC10507596

[3] Sinka L Abraira L Imbach LL Zieglgansberger D Santamarina E Alvarez-Sabin J Ferreira-Atuesta C Katan M Scherrer N Bicciato G. Association of mortality and risk of epilepsy with type of acute symptomatic seizure after ischemic stroke and an updated prognostic model. JAMA Neurol. 2023;80:605–613. doi: 10.1001/jamaneurol.2023.0611 37036702 10.1001/jamaneurol.2023.0611 PMC10087089

[4] Beghi E D’Alessandro R Beretta S Consoli D Crespi V Delaj L Gandolfo C Greco G La Neve A Manfredi M; Epistroke Group. Incidence and predictors of acute symptomatic seizures after stroke. Neurology. 2011;77:1785–1793. doi: 10.1212/WNL.0b013e3182364878 21975208 10.1212/WNL.0b013e3182364878

[5] Fisher RS Acevedo C Arzimanoglou A Bogacz A Cross JH Elger CE Engel J Jr Forsgren L French JA Glynn M. ILAE official report: a practical clinical definition of epilepsy. Epilepsia. 2014;55:475–482. doi: 10.1111/epi.12550 24730690 10.1111/epi.12550

[6] Galovic M Dohler N Erdelyi-Canavese B Felbecker A Siebel P Conrad J Evers S Winklehner M von Oertzen TJ Haring HP. Prediction of late seizures after ischaemic stroke with a novel prognostic model (the SeLECT score): a multivariable prediction model development and validation study. Lancet Neurol. 2018;17:143–152. doi: 10.1016/S1474-4422(17)30404-0 29413315 10.1016/S1474-4422(17)30404-0

[7] Herzig-Nichtweiss J Salih F Berning S Malter MP Pelz JO Lochner P Wittstock M Gunther A Alonso A Fuhrer H. Prognosis and management of acute symptomatic seizures: a prospective, multicenter, observational study. Ann Intensive Care. 2023;13:85. doi: 10.1186/s13613-023-01183-0 37712992 10.1186/s13613-023-01183-0 PMC10504169

[8] Hesdorffer DC Benn EK Cascino GD Hauser WA. Is a first acute symptomatic seizure epilepsy? Mortality and risk for recurrent seizure. Epilepsia. 2009;50:1102–1108. doi: 10.1111/j.1528-1167.2008.01945.x 19374657 10.1111/j.1528-1167.2008.01945.x

[9] Trinka E Cock H Hesdorffer D Rossetti AO Scheffer IE Shinnar S Shorvon S Lowenstein DH. A definition and classification of status epilepticus--report of the ILAE task force on classification of status epilepticus. Epilepsia. 2015;56:1515–1523. doi: 10.1111/epi.13121 26336950 10.1111/epi.13121

[10] Leitinger M Trinka E Gardella E Rohracher A Kalss G Qerama E Hofler J Hess A Zimmermann G Kuchukhidze G. Diagnostic accuracy of the Salzburg EEG criteria for non-convulsive status epilepticus: a retrospective study. Lancet Neurol. 2016;15:1054–1062. doi: 10.1016/S1474-4422(16)30137-5 27571157 10.1016/S1474-4422(16)30137-5

[11] Hirsch LJ Fong MWK Leitinger M LaRoche SM Beniczky S Abend NS Lee JW Wusthoff CJ Hahn CD Westover MB. American clinical neurophysiology society’s standardized critical care EEG terminology: 2021 version. J Clin Neurophysiol. 2021;38:1–29. doi: 10.1097/WNP.0000000000000806 33475321 10.1097/WNP.0000000000000806 PMC8135051

[12] Fisher RS Cross JH French JA Higurashi N Hirsch E Jansen FE Lagae L Moshe SL Peltola J Roulet Perez E. Operational classification of seizure types by the International League Against Epilepsy: position paper of the ILAE commission for classification and terminology. Epilepsia. 2017;58:522–530. doi: 10.1111/epi.13670 28276060 10.1111/epi.13670

[13] Tibshirani R. The lasso method for variable selection in the Cox model. Stat Med. 1997;16:385–395. doi: 10.1002/(sici)1097-0258(19970228)16:4<385::aid-sim380>3.0.co;2-3 9044528 10.1002/(sici)1097-0258(19970228)16:4<385::aid-sim380>3.0.co;2-3

[14] Harrell FE Jr Lee KL Mark DB. Multivariable prognostic models: issues in developing models, evaluating assumptions and adequacy, and measuring and reducing errors. Stat Med. 1996;15:361–387. doi: 10.1002/(SICI)1097-0258(19960229)15:4<361::AID-SIM168>3.0.CO;2-4 8668867 10.1002/(SICI)1097-0258(19960229)15:4<361::AID-SIM168>3.0.CO;2-4

[15] Schrag A Siddiqui UF Anastasiou Z Weintraub D Schott JM. Clinical variables and biomarkers in prediction of cognitive impairment in patients with newly diagnosed Parkinson’s disease: a cohort study. Lancet Neurol. 2017;16:66–75. doi: 10.1016/S1474-4422(16)30328-3 27866858 10.1016/S1474-4422(16)30328-3 PMC5377592

[16] Backes D Rinkel GJE Greving JP Velthuis BK Murayama Y Takao H Ishibashi T Igase M terBrugge KG Agid R. ELAPSS score for prediction of risk of growth of unruptured intracranial aneurysms. Neurology. 2017;88:1600–1606. doi: 10.1212/WNL.0000000000003865 28363976 10.1212/WNL.0000000000003865

[17] Zabor EC Gonen M Chapman PB Panageas KS. Dynamic prognostication using conditional survival estimates. Cancer. 2013;119:3589–3592. doi: 10.1002/cncr.28273 23913639 10.1002/cncr.28273

[18] Bonnett LJ Tudur-Smith C Williamson PR Marson AG. Risk of recurrence after a first seizure and implications for driving: further analysis of the Multicentre study of early Epilepsy and Single Seizures. BMJ. 2010;341:c6477. doi: 10.1136/bmj.c6477 21147743 10.1136/bmj.c6477 PMC2998675

[19] Brown JW Lawn ND Lee J Dunne JW. When is it safe to return to driving following first-ever seizure? J Neurol Neurosurg Psychiatry. 2015;86:60–64. doi: 10.1136/jnnp-2013-307529 24769470 10.1136/jnnp-2013-307529

[20] Schmedding E Darde JB Gappmeier B Kirker J Kraemer G Markschies N Ojala M Sundqvist A Valdes E Vespigniani H. Epilepsy and driving in Europe. Second European Working Group on Epilepsy and Driving; 2005.

[21] Pitkanen A Roivainen R Lukasiuk K. Development of epilepsy after ischaemic stroke. Lancet Neurol. 2016;15:185–197. doi: 10.1016/S1474-4422(15)00248-3 26597090 10.1016/S1474-4422(15)00248-3

[22] Engel J Jr. Concepts of epilepsy. Epilepsia. 1995;36(Suppl 1):S23–S29. doi: 10.1111/j.1528-1157.1995.tb01648.x 23057107 10.1111/j.1528-1157.1995.tb01648.x

[23] Chou CC Shih YC Chiu HH Yu HY Lee IH Lin YY Lee CC Peng SJ. Strategic infarct location for post-stroke seizure. Neuroimage Clin. 2022;35:103069. doi: 10.1016/j.nicl.2022.103069 35689977 10.1016/j.nicl.2022.103069 PMC9190039

[24] Schaper FLWVJ Nordberg J Cohen AL Lin C Hsu J Horn A Ferguson MA Siddiqi SH Drew W Soussand L. Mapping lesion-related epilepsy to a human brain network. JAMA Neurol. 2023;80:891–902. doi: 10.1001/jamaneurol.2023.1988 37399040 10.1001/jamaneurol.2023.1988 PMC10318550

[25] Troscher AR Gruber J Wagner JN Bohm V Wahl AS von Oertzen TJ. Inflammation mediated epileptogenesis as possible mechanism underlying ischemic post-stroke epilepsy. Front Aging Neurosci. 2021;13:781174. doi: 10.3389/fnagi.2021.781174 34966269 10.3389/fnagi.2021.781174 PMC8711648

[26] Ben-Ari Y Dudek FE. Primary and secondary mechanisms of epileptogenesis in the temporal lobe: there is a before and an after. Epilepsy Curr. 2010;10:118–125. doi: 10.1111/j.1535-7511.2010.01376.x 20944823 10.1111/j.1535-7511.2010.01376.x PMC2951692

[27] Kadam SD White AM Staley KJ Dudek FE. Continuous electroencephalographic monitoring with radio-telemetry in a rat model of perinatal hypoxia-ischemia reveals progressive post-stroke epilepsy. J Neurosci. 2010;30:404–415. doi: 10.1523/JNEUROSCI.4093-09.2010 20053921 10.1523/JNEUROSCI.4093-09.2010 PMC2903060

[28] Shen Y Gong Y Ruan Y Chen Z Xu C. Secondary epileptogenesis: common to see, but possible to treat? Front Neurol. 2021;12:747372. doi: 10.3389/fneur.2021.747372 34938259 10.3389/fneur.2021.747372 PMC8686764

[29] Chen TS Huang TH Lai MC Huang CW. The role of glutamate receptors in epilepsy. Biomedicines. 2023;11:783. doi: 10.3390/biomedicines11030783 36979762 10.3390/biomedicines11030783 PMC10045847

[30] Ferreira-Atuesta C Dohler N Erdelyi-Canavese B Felbecker A Siebel P Scherrer N Bicciato G Schweizer J Sinka L Imbach LL. Seizures after ischemic stroke: a matched multicenter study. Ann Neurol. 2021;90:808–820. doi: 10.1002/ana.26212 34505305 10.1002/ana.26212 PMC9292028

[31] Doerrfuss JI Holtkamp M Vorderwulbecke BJ. The SeLECT 2.0 score-significance of treatment with antiseizure medication. JAMA Neurol. 2023;80:1252. doi: 10.1001/jamaneurol.2023.3371 10.1001/jamaneurol.2023.3371 37747712

[32] Schubert KM Sinka L Galovic M. The SeLECT 2.0 score-significance of treatment with antiseizure medication-reply. JAMA Neurol. 2023;80:1252–1253. doi: 10.1001/jamaneurol.2023.3374 10.1001/jamaneurol.2023.3374 37747727

[33] Koepp MJ Trinka E Mah YH Bentes C Knake S Gigli GL Serratosa JM Zelano J Magalhães LM Pereira A. Antiepileptogenesis after stroke-trials and tribulations: methodological challenges and recruitment results of a Phase II study with eslicarbazepine acetate. Epilepsia Open. 2023;8:1190–1201. doi: 10.1002/epi4.12735 36944588 10.1002/epi4.12735 PMC10472381

[34] Koepp M Trinka E Loscher W Klein P. Prevention of epileptogenesis - are we there yet [published online February 13, 2024]? Curr Opin Neurol. 2024;37. doi: 10.1097/WCO.0000000000001256 10.1097/WCO.0000000000001256 38345421

[35] Labovitz DL Hauser WA Sacco RL. Prevalence and predictors of early seizure and status epilepticus after first stroke. Neurology. 2001;57:200–206. doi: 10.1212/wnl.57.2.200 11468303 10.1212/wnl.57.2.200

[36] Cordonnier C Henon H Derambure P Pasquier F Leys D. Influence of pre-existing dementia on the risk of post-stroke epileptic seizures. J Neurol Neurosurg Psychiatry. 2005;76:1649–1653. doi: 10.1136/jnnp.2005.064535 16291888 10.1136/jnnp.2005.064535 PMC1739446

[37] Meletti S Cuccurullo C Orlandi N Borzì G Bigliardi G Maffei S Del Giovane C Cuoghi Costantini R Giovannini G Lattanzi S. Prediction of epilepsy after stroke: proposal of a modified SeLECT 2.0 score based on posttreatment stroke outcome. Epilepsia. 2024;65:3234–3243. doi: 10.1111/epi.18114 39235830 10.1111/epi.18114

